# Food Adulterations

**Published:** 1887-07

**Authors:** 


### FOOD ADULTERATION.

The New York *World*, whose enterprise embraces almost everything moral, religious, political and economical, has recently turned its attention to the evil of food adulteration. Out of one hundred samples of tea collected of retail dealers, twelve were found to be adulterated, for the most part, with foreign leaves, known as *lie* tea. Of one hundred samples of ground coffee, twenty-eight were mixed with chicory and peas. Of one hundred samples of sugar, only two were found to be adulterated with glucose starch, so that of the three hundred samples tested, forty-two, or an average of fourteen per cent, were found to be impure, enough in all conscience to show the necessity of local legislation, sufficiently severe to effectually put an end to these abominable and thoroughly dishonest practices.

